# Blood DNA methylation of *CRF* and its association with amygdala volume and mood in Cushing’s syndrome

**DOI:** 10.3389/freae.2024.1435634

**Published:** 2024-10-02

**Authors:** Richard S. Lee, Alicia Santos, Henri Garrison-Desany, Anna Aulinas, Jenny L. Carey, Yolanda Vives-Gilabert, Olivia H. Cox, Gabriel Cuilan, Susan M. Webb, Eugenia Resmini

**Affiliations:** ^1^ Department of Psychiatry, Johns Hopkins University School of Medicine, Baltimore, MD, United States; ^2^ Department of Endocrinology, Hospital de la Santa Creu I Sant Pau, Research Center for Pituitary Diseases, Institut de Recerca Sant Pau (IR-Sant Pau), EndoERN, CIBERER Unit 747, ISCIII, Barcelona, Spain; ^3^ Department of Medicine, Universitat Autonoma de Barcelona, Barcelona, Spain; ^4^ Department of Mental Health, Johns Hopkins Bloomberg School of Public Health, Baltimore, MD, United States; ^5^ Department of Medicine, Universitat de Vic-Universitat Central de Catalunya, Vic, Spain; ^6^ Intelligent data analysis laboratory (IDAL), Electronic Engineering Department, Escuela técnica superior de ingeniería (ETSE), Universidad de Valencia (UV), Valencia, Spain

**Keywords:** Cushing’s syndrome, hypercortisolism, corticotropin-releasing factor, DNA methylation, amygdala

## Abstract

**Objective:**

The impact of chronic exposure to stress or glucocorticoids on psychiatric symptoms has been exemplified by cases of iatrogenic or endogenous hypercortisolism such as Cushing’s syndrome (CS). The amygdala plays an important role in mediating both stress and affective responses, and one of the key factors that link stress response and psychiatric symptoms is the corticotropin-releasing factor (CRF). Epigenetic changes, especially those occurring on CpG dinucleotides in DNA of glucocorticoid target genes in blood, have been previously implicated as potential predictors of glucocorticoid-related events in the central nervous system (CNS). In this study, we examined amygdala volume and mood symptoms in CS patients and aimed at evaluating whether these parameters were associated with blood DNA methylation of *CRF*.

**Methods:**

In this cross-sectional study, 32 CS patients and 32 healthy controls matched for age, sex, and years of education underwent an MRI scan, a Beck Depression Inventory-II, and a State-Trait Anxiety Inventory. Genomic DNA extracted from total leukocytes were used for DNA methylation analysis of several CpG dinucleotides at the *CRF* promoter region.

**Results:**

Significant associations between *CRF* methylation vs. amygdala volume (CpG-1, *P* = 0.006) and depression scores (CpG-2, *P* = 0.01) were found. To assess whether the promoter CpG methylation has functional consequences, we examined RNA and DNA extracted from non-CS, postmortem amygdala tissues. A significant association between CpG methylation and gene expression (CpG-1, *P* = 0.004) was observed.

**Conclusion:**

These results demonstrate that methylation levels of the *CRF* promoter CpGs are associated with amygdala volume in CS and related mood symptoms. Methylation levels may also be associated with *CRF* expression. This finding supports the feasibility of using epigenetic patterns in blood as a surrogate for assessing GC-related pathologies in the brain.

## Introduction

Cushing’s syndrome (CS) is a rare disease caused by chronic glucocorticoid (GC) excess. It is characterized by central obesity, moon face, muscle weakness, red or purple striae, easy bruising, bone loss, hypertension, fatigue, emotional lability, anxiety, and depression (Starkman et al., 1981; Sonino and Fava, 2001; Arnaldi et al., 2003; Nieman et al., 2008). While many of the symptoms improve following the resolution of hypercortisolism, other symptoms persist, especially GCs’ deleterious effects on the brain. Several studies have documented cognitive impairments and reduced brain volume in CS patients even months and years after biochemical or surgical cure (Bourdeau et al., 2005; Tiemensma et al., 2010; Resmini et al., 2012; Andela et al., 2015; Santos et al., 2015). These findings highlight the enduring, yet unidentified factors that mediate the effects of chronic hypercortisolism on brain function and justify the use of CS as an appropriate human model for understanding the impact of chronic cortisol and stress exposure in the general population.

Previously, we have demonstrated in a mouse model of CS that hypercortisolism is associated with epigenetic changes in the stress response and mood disorder gene *Fkbp5* (Scammell et al., 2001; Binder et al., 2004; Lee et al., 2010; Attwood et al., 2011; Klengel et al., 2013). These epigenetic changes consist of, but are not limited to, the addition or removal of methyl groups to cytosine and guanine (CpGs) dinucleotide sequence of DNA at regulatory regions such as gene promoters, enhancers, and disease- and tissue-specific regions (Irizarry et al., 2009). At *Fkbp5*, DNA methylation levels were significantly associated with several GC-induced parameters such as increase in adiposity, hyperglycemia, atrophy of specific organs, and changes in behavior (Lee et al., 2011). Specifically, exposure to excess glucocorticoids (GCs) was associated with DNA methylation changes at intronic CpGs located near GC response elements (GREs) within the gene (Lee et al., 2010), and GC-induced CpG methylation assessed at the *Fkbp5* locus in blood correlated significantly with its methylation and expression levels in the brain (Ewald et al., 2014). A more comprehensive, genome-wide study using the same mouse model showed that GC-induced methylation changes detected at a large number of genes in blood also occurred at the same genes in the brain (Seifuddin et al., 2017).

In human CS, we also showed that hypercortisolism is associated with the reduction of intronic DNA methylation of *FKBP5* in blood, which in turn correlated with memory impairment and hippocampal volume (Resmini et al., 2016). Since GCs have been shown to site-specifically alter DNA methylation and gene function, genes that are targeted by GCs and play a mechanistic role in CS pathology may be good candidates as peripheral biomarkers that can reflect physiological processes occurring in the central nervous system (CNS).

An important neuroendocrine factor that governs GC-related behaviors and thus can serve as a candidate peripheral biomarker of hypercortisolism is corticotropin-releasing factor (CRF or CRH for corticotropin-releasing hormone). CRF is a neuropeptide that serves as one of the core components of the hypothalamus-pituitary-adrenal (HPA) axis that mediates the body’s stress response. In response to stress exposure, the hypothalamic release of CRF causes the pituitary release of ACTH (adrenocorticotropic hormone), which culminates in the adrenal release of cortisol. Due to its involvement in the HPA axis and abundant expression in the brain, CRF also plays a central role in stress-related psychiatric disorders. CRF hyperactivity is often associated with depression and anxiety, and exposure to early-life stress serves as a risk factor for anxiety and mood disorders by causing persistent dysregulation of *CRF* (Arborelius et al., 1999; Heim and Nemeroff, 2001). In addition, CRF can contribute to negative emotional states that can drive cycles of drug addiction and relapse (Koob, 1999).

Importantly, it is thought that CRF signaling beyond its direct role within the hypothalamus-pituitary-adrenal (HPA) axis such as in the amygdala (Hostetler and Ryabinin, 2013) may be responsible for some of the GC-borne mood disorder symptoms such as those observed in CS. In fact, reduction in amygdala volume has been observed in CS patients and associated with alterations in mood symptoms (Santos et al., 2017). In mice, stress exposure and neurodevelopmental alterations involve epigenetic mechanisms, namely DNA methylation, in governing *Crf* expression and anxiety-like behaviors (McGill et al., 2006; Elliott et al., 2010). Further, CRF and downstream signaling through its interacting receptor CRFR1 have been shown to play a central role in the amygdala in contextual fear conditioning and anxiety-related behaviors, such as post-traumatic stress disorder (PTSD) (Hollis et al., 2016; Itoga et al., 2016). Therefore, we hypothesize that CRF signaling in the amygdala may be associated with some of the behavioral symptoms observed in CS patients.

In this study, we examined amygdala volume and mood scores in CS patients and aimed at evaluating whether these parameters were associated with blood DNA methylation of the *CRF* gene. A secondary goal of the study was to test the role of DNA methylation in *Crf* expression by using postmortem amygdala tissues from which both DNA methylation and mRNA could be measured. Demonstrating that some of the CS-related pathologies could be linked to blood CRF methylation, similar to what has been reported with *FKBP5* and hippocampal volume, would further support the feasibility of using epigenetic patterns in blood as a surrogate for assessing GC-related pathologies in the brain.

## Materials and methods

### CS patients and controls

In this cross-sectional study, 32 right-handed CS patients clinically followed at the Hospital Sant Pau (Barcelona, Spain), and 32 healthy controls matched for age, sex, and years of education were included. Control subjects were recruited among right-handed healthy volunteers who had previously participated in clinical studies at the Hospital Sant Pau. They had no history of excess GC exposure and were free of medications.

At the time of the study, 9 CS patients were hypercortisolemic (active disease), with 7 cases of pituitary and 2 of adrenal origins. All were on medical therapy, 4 on metyrapone, and 5 on ketoconazole. Four of nine were awaiting surgery (2 adrenal and 2 pituitary). The remaining 5 of the nine had previously undergone unsuccessful pituitary transsphenoidal neurosurgery. Twenty-three patients were in remission (biochemically cured), with 18 cases of pituitary and 5 of adrenal origins. All cured CS of pituitary origin had undergone transsphenoidal surgery (12) or pituitary radiotherapy (6). Unilateral adrenalectomy was performed in the 5 CS patients of adrenal origin. Four patients had adrenal insufficiency at the time of the study and required hydrocortisone replacement therapy, whereas the remaining 19 did not require chronic substitution therapy.

CS was considered in remission if patients achieved adrenal insufficiency or morning cortisol suppression (<50 nmol/L; <1.8 μg/dL) after 1 mg dexamethasone overnight and repeatedly normal 24-h urinary free cortisol levels (measured by radioimmunoassay following urine extraction with an organic solvent; normal <280 nmol/24 h). The mean time of biochemical cure at the study date was 7.1 ± 2.2 years (mean ± STD). Patients exhibited poorer verbal and visual memory performance than controls, as previously described (Resmini et al., 2012). The duration of hypercortisolism was considered as the time from symptom onset until remission of hypercortisolism after treatment and was assessed by the endocrinologist in charge. At diagnosis, the duration of hypercortisolism was estimated by personal interview, detailed review of medical records, and photographs of patients. All information was written or kept in clinical records, together with data regarding the achievement of the biochemical cure. The mean duration of hypercortisolism was 5.1 ± 2.8 years. CS patients with diabetes mellitus and growth hormone deficiency were excluded since cognitive deficits and brain volume reductions have also been described in these conditions (den Heijer et al., 2003; Popovic et al., 2004; Gold et al., 2007; Bruehl et al., 2009). All patients and controls signed an informed consent after study approval by the Hospital Ethics Committee. None of the participants had a past medical history of head injury, cerebrovascular disease, psychiatric disorders, or use of tranquilizers.

### Mood scores and MRI

Beck Depression Inventory-II (BDI-II) is a self-reported measure of the severity of depressive symptoms (Beck and Beamesderfer, 1974; Beck et al., 1996). It has 21 items with a four-point scale ranging from 0 to 3. The total score is the sum of each item rating, ranging from 0 to 63. The manual states that higher scores indicate more severe depressive symptoms. Scores 0–13 indicate minimal depression, 14–19 indicate mild depression, 20–28 indicate moderate depression, and 29–63 indicate severe depression. State-Trait Anxiety Inventory (STAI) is a self-reported measure that includes two subscales to evaluate two types of anxiety: state anxiety (anxiety related to current events) and trait anxiety (anxiety as a personal characteristic) (Spielberger et al., 1983). Each subscale has 20 questions with a four-point scale ranging from 0 to 3. The total score for each subscale is the sum of each item rating and can range from 0 to 60. Higher scores indicate higher levels of anxiety. MRI was obtained using a 3-Tesla Philips Achieva facility (software version 2.1.3.2). All volumetric scores were obtained and normalized to the estimated intracranial volume of each individual, as previously described (Resmini et al., 2012; Santos et al., 2017).

### Postmortem amygdala samples

Postmortem amygdala tissues from controls (N = 20) without any psychiatric disorders were obtained from the Maryland Psychiatric Research Center. The cohort demographics are as follows: 43.8 ± 14.7 years of age; 17 Whites, 2 Blacks, and 1 Hispanic; and 13 females and 7 males. The purpose of these samples was to examine the relationship between promoter methylation and expression of *CRF*.

### Genomic DNA extraction from total leukocytes and brain tissues

Genomic DNA extraction from total leukocytes was carried out using an adapted proteinase K and phenol protocol in all CS and age-matched control subjects (Sambrook et al., 1989). Blood samples from patients were collected in EDTA tubes to reduce blood clotting and DNA degradation. White blood cells were isolated from the buffy coat, pelleted, and stored at −80°C. Genomic DNA was isolated from the white blood cell pellet using a buffer containing 0.1 M MgCl_2_, 0.02 M EDTA, 0.5% SDS, 0.01 M Tris, pH 8.0, and 1 mg/mL of proteinase K at 37°C overnight. The lysates were homogenized by passing through a blunt 20-gauge needle (0.9 mm diameter) at 4°C, and DNA was purified by phenol:chloroform:isoamyl alcohol (25:24:1) extraction followed by ethanol precipitation. For the postmortem amygdala tissues, DNA was extracted using the Allprep DNA/RNA Micro Kit according to the manufacturer’s instructions (Qiagen, Germantown, MD). Finally, genomic DNA from blood and brain was resuspended or eluted in Tris–EDTA buffer and quantified by Qubit Fluorometer (ThermoScientific, Waltham, MA).

### Bisulfite PCR and pyrosequencing

DNA methylation was measured by pyrosequencing of the PCR products, which measures methylation variation at >90% precision (Colella et al., 2003). Two hundred and fifty ng of DNA was bisulfite-converted using the EZ DNA Methylation-Gold kit, according to the manufacturer’s protocol (Zymo Research, Irvine, CA). Two sets of primers were designed to amplify a 248-bp promoter region of the human *CRF* gene:

Outside – A: TTAATGGATTTGGTTTTT GAGTT;
Outside – B: ACA​CAC​CCC​TAA​TAT​AAC​CTT​TCA;
Nested – A: TTTAGAGTTT GGAGTGGGATT; and
Nested – B: AAA​CCC​TTC​CAT​TTT​AAA​ACT​C.

The genomic organization of *CRF* and three promoter CpG dinucleotides targeted by bisulfite pyrosequencing are shown in Figure 1. The human genomic sequence that harbors the three CpGs, including 20 basepairs flanking both sides of the CpGs, share 100% sequence identity with those of the mouse and rat. Genomic coordinates of the three consecutive CpGs are CpG-1 (67,090,798), CpG-2 (67,090,792), and CpG-3 (67,090,776) on chromosome 8q13.1, according to the UCSC Genome Browser GRCh37/hg19 assembly. Further, the CpGs are functionally significant, based on their proximity to a putative glucocorticoid response element and a previous study implicating them to stress-induced changes in methylation (Elliott et al., 2010). Thermocycling was carried out using the Veriti thermal cycler (Life Technologies, Carlsbad, CA), and 25 ng of bisulfite-treated DNA was used for each PCR reaction. An additional nested PCR was performed with 2 µL of the previous PCR reaction and biotinylated Nested–A primer (Nested–B being unmodified). Amplification for both PCR steps consisted of 40 cycles (94°C for 1 min, 53°C for 30 s, 72°C for 1 min). PCR products were confirmed on agarose gels. Pyro Gold reagents were used to prepare samples for pyrosequencing according to the manufacturer’s instructions (Qiagen). For each sample, the biotinylated PCR product was mixed with streptavidin-coated sepharose beads (GE Healthcare, Waukesha, WI), binding buffer, and Milli-Q water and shaken at room temperature. A vacuum prep tool was used to isolate the sepharose bead-bound single-stranded PCR products. The attached DNAs were released into a PSQ HS 96-plate containing pyrosequencing primer (*CRF* Pyro: CCT​ATA​ATT​TAT​ACA​AAA​AC) in annealing buffer. Pyrosequencing reactions were performed on the PyroMark MD System (Qiagen). CpG methylation quantification was performed with the Pyro Q-CpGt 1.0.9 software (Qiagen). An internal quality-control step was employed to disqualify any assays that contained unconverted DNA. The percentage of methylation at each CpG, as determined by pyrosequencing, was compared among DNA samples from Control, cured CS and active CS patients.

**FIGURE 1:**
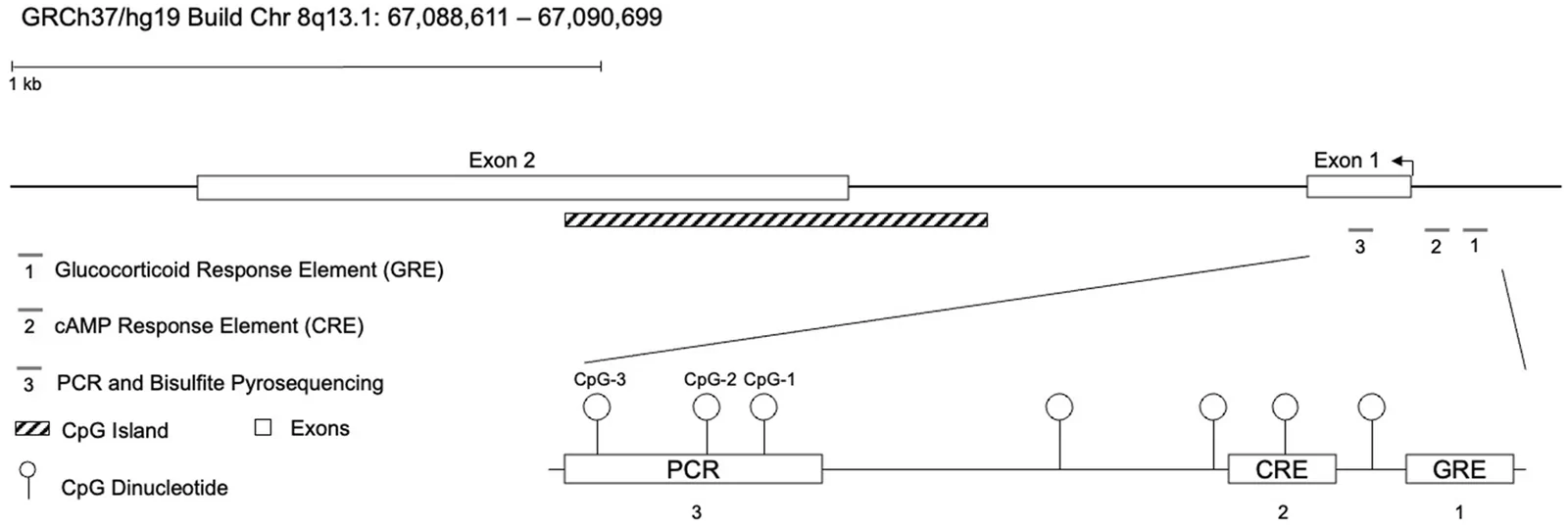
Genomic organization of the corticotropin-releasing factor (*CRF*) locus. The *CRF* gene is 2.2 kilobases in size and consists of 2 exons. It is located on the negative strand of human chromosome 8q13.1 (UCSC: GRCh37/hg19 assembly). The diagram depicts the promoter and 5′-UTR CpGs assayed, as well as two putative cAMP- and glucocorticoid-response elements.

### RNA extraction and quantitative real-time PCR (qPCR)

RNA was extracted from ∼25 mg of postmortem amygdala samples using the Allprep DNA/RNA Micro Kit according to the manufacturer’s instructions (Qiagen). The total RNA eluted from the Allprep columns was quantified by TapeStation 2.0 bioanalyzer (Agilent Technologies, Santa Clara, CA), and 5 out of 25 samples with RIN numbers less than 7 were excluded from gene expression analysis. The QuantiTect Reverse Transcription Kit (Qiagen) was used to generate cDNA for quantitative real-time PCR. *CRF* expression analysis was carried out in triplicate using 1X Taqman Universal Master Mix (Applied Biosystems, Foster City, CA), 1 μL Taqman probes for *CRF* or house-keeping gene β-actin (*ACTB*) for normalization, and 30 ng of cDNA template in a total volume of 20 μL. Real-time reactions were performed on an Applied Biosystems QuantStudio 5 real-time PCR system with standard PCR conditions (50°C for 2 min; 95°C for 10 min; and 95°C for 15 s and 60°C for 1 min for 40 cycles). Each set of triplicates was checked to ensure that the threshold cycle (Ct) values were all within 0.5 Ct of each other. To determine relative expression values, the -ΔΔCt method was used (Applied Biosystems).

### Statistical analysis

Data were analyzed using the statistical software Stata 15.1 (StataCorp, 2017; R Core Team, 2018). *P*-values were corrected for multiple testing across methylation sites using a Bonferroni correction for each CpG locus, resulting in an adjusted cutoff for significance of 0.02. An exploratory analysis was conducted between CS cases (active and cured) and controls to assess potential confounders to the comparability of cases and controls. Major sociodemographic factors, including age, gender, and education, as well as biological and mental health characteristics, such as amygdala volume and scores on the BDI-II and STAI, were tested for differences, with crude associations calculated using Student’s t-test for quantitative traits and Chi-squared tests for categorical traits.

Association between *CRF* promoter methylation across the three CpG sites and CS case status was conducted using both crude Pearson’s correlation adjusting for age, gender, and education level of participants and linear regression adjusting for age, gender, and years of education. Given the tendency of nearby CpG dinucleotides to be similarly methylated (Martin et al., 2015), the methylation values across the three CpGs were averaged, and the regression was run again as a sensitivity analysis of global methylation in the region. Then, the difference in methylation between active CS cases only and healthy controls was examined with linear regression, as well as differences between active CS cases and cured CS cases to examine any effects treatment may have on methylation status.

The relationship between *CRF* methylation and mental health metric scores was also assessed using Pearson’s correlation and adjusted linear regression. This was repeated for the state and trait anxiety (STAI) subscales as a sensitivity analysis. Then, *CRF* methylation and amygdala volume were examined using similar statistical tests as described above, using right, left, and total amygdala volumes as the outcome of interest for the linear regression analysis.

Finally, using post-mortem amygdala tissues from healthy individuals, DNA methylation was assessed against gene expression levels of *CRF*, and Pearson’s correlation was calculated between relative *CRF* expression and each CpG site.

## Results

### Sociodemographic and biometric data for CS patients and controls

Exploratory analysis found no statistically significant differences between CS cases and healthy controls on major sociodemographic and clinical variables. The mean age of controls was 43.8 ± 11.0 years compared to 44.5 ± 11.6 years for CS (*P* = 0.79, Table 1). Gender distributions were also similar, with 21.9% (N = 7) of controls and 18.8% (N = 6) of CS cases being males (*P* = 0.23). Differences in the years of education attained was 0.6 years (*P* = 0.45). Amygdala volumes showed no significant differences between controls and CS, although CS volumes were somewhat smaller: right amygdala volume (2167.8 ± 255.8 vs. 2050.7 ± 366.4 cm^3^, *P* = 0.14); left (1865.4 ± 254.1 vs. 1871.5 ± 301.0 cm^3^, *P* = 0.93); and total (4033.2 ± 485.3 vs. 3922.3 ± 642.6 cm^3^, *P* = 0.44). It should be noted that when comparing active CS cases and controls, mean difference in the right amygdala volume was borderline significant (2167.8 ± 255.8 vs. 1944.2 ± 501.6 cm^3^, *P* = 0.073). In contrast, all three mental health scores showed highly significant associations via the t-test, with the BDI scores in controls on average 2.6 ± 3.5 and 9.3 ± 5.6 among CS cases (*P* < 0.0001). The state score for anxiety had a difference of 9.5 (*P* = 0.0007), and the trait score had a difference of 13.3 between controls and CS (*P* < 0.0001).

**TABLE 1:** Demographics, amygdala volumes, and affective symptom scores in CS patients and controls.

	Controls (N = 32)	Cushing’s syndrome (N = 32)	*P*-value
Age (yrs)	43.8 (11.0)	44.5 (11.6)	0.79
Sex (Male: Female)	7:25	6:26	0.23
Education (yrs)	13.8 (3.6)	13.2 (3.5)	0.45
Right Amg. Vol. (cm^3^)	2,167.8 (255.8)	2,050.7 (366.4)	0.14
Left Amg. Vol. (cm^3^)	1,865.4 (254.1)	1,871.5 (301.0)	0.93
Total Amg. Vol. (cm^3^)	4,033.2 (485.3)	3,922.3 (642.6)	0.44
BDI	2.6 (3.5)	9.3 (5.6)	2.7 × 10^-7^
STAI – State	10.2 (7.6)	19.7 (12.9)	7.0 × 10^-4^
STAI – Trait	11.2 (6.6)	24.5 (11.3)	2.7 × 10^-7^

Data are presented as mean (standard deviation). *P*-values reflect statistical significance between healthy controls and Cushing’s syndrome that includes both active and cured cases. *P*-values for quantitative traits are derived from Student’s t-test. *P*-values from categorical traits are derived from Chi^2^ test.

### CRF promoter methylation in CS patients and controls

We determined DNA methylation levels of three stress-relevant, consecutive CpG dinucleotides at the *CRF* promoter (Figure 1) in the CS patients and healthy controls. Bisulfite pyrosequencing showed a trend of reduced methylation in CS samples across all three CpGs, with only CpG-3 achieving statistical significance: CpG-1 (−2.5%, *P* = 0.18); CpG-2 (−1.6%, *P* = 0.39), and CpG-3 (−2.9%, *P* = 0.009) (Figure 2A). Adjusting for common sociodemographic factors (age, gender, and education), similar effects were observed: CpG-1 (−2.5%, *P* = 0.15); CpG-2 (−1.7%, *P* = 0.33), CpG-3 (−3.1%, *P* = 0.002) (Table 2). Mean methylation levels across the three CpGs between the groups were also significantly different (−2.3%, *P* = 0.04). CS samples further stratified into active vs. cured status showed consistent reduction in the active CS compared to cured CS samples across all three CpGs, but none of the reductions were significant (methylation difference <3.7%, *P* > 0.27). Notably, CpG-3 showed differences in methylation between active CS vs. controls (−3.5%, *P* = 0.03) and cured CS vs. controls (−2.7%, *P* = 0.02) (Figure 2B). Examining only the active CS cases vs. healthy controls and adjusting for age, gender, and education showed similar patterns as active and cured CS compared to controls: CpG-1 (−2.0%, *P* = 0.27), CpG-2 (−0.6%, *P* = 0.75), and CpG-3 (−2.91%, *P* = 0.006).

**FIGURE 2:**
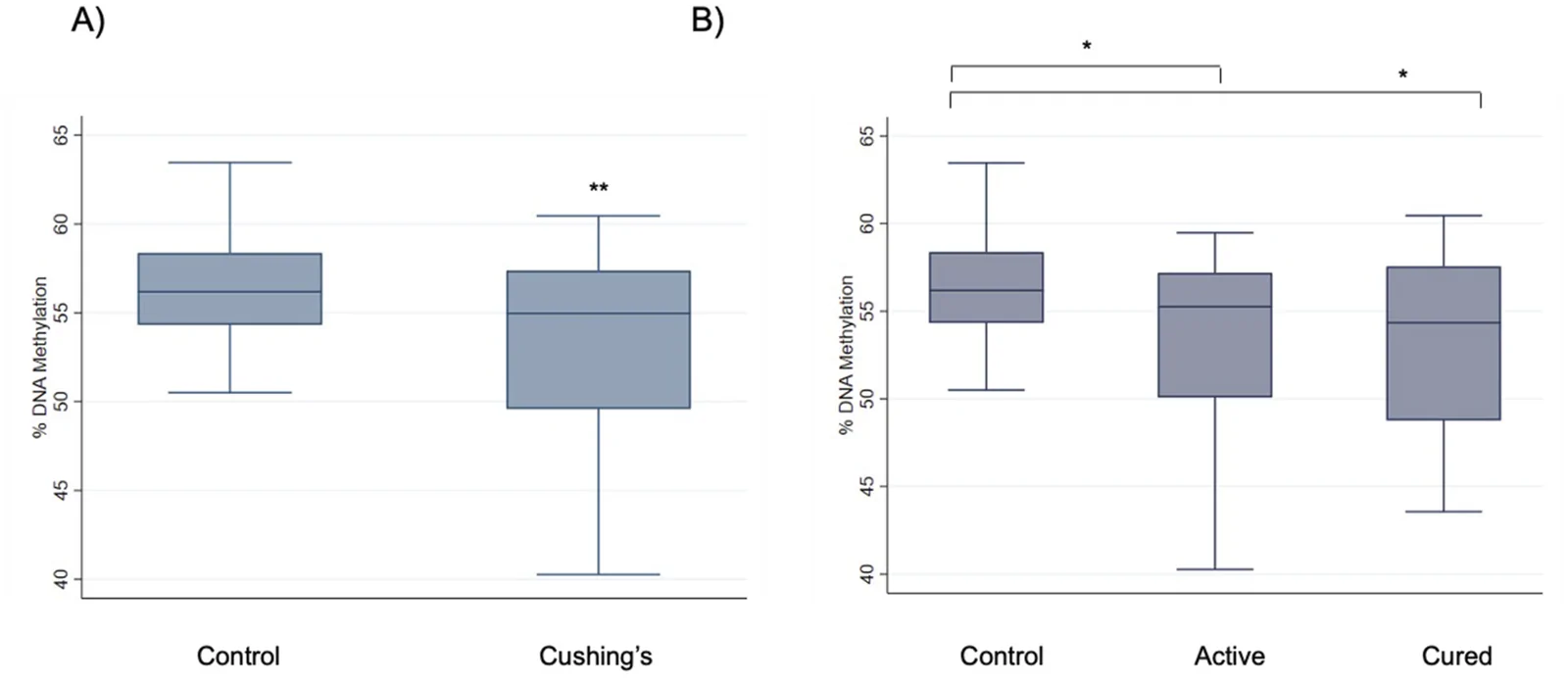
Reduction of *CRF* methylation in Cushing’s syndrome. **(A)** The boxplot shows a comparison of methylation differences between healthy controls (N = 32) and CS patients (N = 32) at the *CRF* promoter CpG-3 in leukocytes. The CS patients include both active (N = 9) and cured patients (N = 23). **(B)** CpG-3 methylation levels were also analyzed after case samples were further segregated into active CS vs. cured CS. *P < 0.05 and **P < 0.01 using Student’s t-test.

**TABLE 2:** Individual and mean *CRF* methylation across three CpG sites vs. CS case status, depression scores (assessed continuously and dichotomously), and amygdala volumes (assessed as total, left, and right amygdala volumes), adjusting for age, sex, and education.

	Cushing’s syndrome case^a^		Beck depression Inventory-II (BDI-II) score^b^				Amygdala volume (cm^3^)^c^					
			BDI continuous score		BDI qualitative score		Total volume		Left volume		Right volume	
	Adjusted coefficient (95% CI)	*P*-value	Adjusted coefficient (95% CI)	*P*-value	Adjusted coefficient (95% CI)	*P*-value	Adjusted coefficient (95% CI)	*P*-value	Adjusted coefficient (95% CI)	*P*-value	Adjusted coefficient (95% CI)	*P*-value
Mean CpG	−2.45 (−4.76, −0.13)	**0.04**	**−0.31 (-0.52, -0.11)**	**0.003**	**0.81 (0.67, 0.98)**	**0.03**	26.76 (−3.39, 56.91)	0.08	12.09 (−2.54, 26.71)	0.10	14.68 (−2.47, 31.82)	0.09
CpG1	−2.52 (−5.97, 0.92)	0.15	−0.20 (−0.40, 0.004)	0.06	0.94 (0.84, 1.04)	0.23	**28.20 (8.35, 48.05)**	**0.006**	**11.87 (2.12, 21.63)**	**0.02**	**16.33 (5.09, 27.57)**	**0.005**
CpG2	−1.71 (−5.17, 1.75)	0.33	**−0.26 (-0.46, -0.06)**	**0.01**	**0.86 (0.76, 0.97)**	**0.02**	5.33 (−15.92, 26.58)	0.62	3.16 (−7.10, 13.42)	0.54	2.18 (−9.90, 14.25)	0.72
CpG3	−3.11 (−5.00, −1.22)	**0.002**	**−0.45 (-0.79, -0.10)**	**0.01**	0.84 (0.70, 1.03)	0.09	11.92 (−24.09, 47.93)	0.51	5.74 (−11.67, 23.15)	0.51	6.18 (−14.28, 26.63)	0.55

Note: Statistically significant associations are highlighted in bold.

^a^ Cushing’s case status was used as an outcome with *CRF*, methylation being used as the predictor in logistic regression.

^b^ Depression was the outcome with *CRF* methylation as a predictor in linear regression for continuous BDI score and logistic regression for qualitative scoring (using score ≥ 20 points).

^c^ Amygdala volume was used as an outcome with *CRF* methylation as a predictor. All models were adjusted for age (years), gender (female), and education (years).

### Association between *CRF* methylation and mood data

We then evaluated whether the methylation levels correlated with data obtained from the Beck Depression Inventory (BDI-II) and State-Trait Anxiety Inventory (STAI). Significant correlations were observed across CpG-2 (r = −0.29, *P* = 0.01) and CpG-3 (r = −0.23, *P* = 0.01), and borderline correlation was observed at CpG-1 (r = −0.22, *P* = 0.06) when *CRF* methylation was compared against BDI-II scores. Linear regression analysis was also performed, and results for CpG-2 vs. BDI-II scores is shown in Figure 3A. In linear regression analysis adjusting for sociodemographic variables, BDI-II scores were significantly associated with CpG-2 (−0.26, *P* = 0.01) and CpG-3 (−0.45, *P* = 0.01), while their association with CpG-1 had a point estimate (−0.20) and *P*-value (*P* = 0.06) similar to those prior to adjustment (Table 2). In contrast, none of the CpGs were significantly associated with neither the STAI Trait (|r|<0.16, *P* > 0.20) nor the STAI State (|r|<0.19, *P* > 0.12) scores. Figure 3B shows the linear regression analysis between CpG-2 methylation levels and STAI State scores. Using linear regression and adjusting for age, education, and gender, the STAI State subscale scores were only significantly associated with CpG-2 (−0.41, *P* = 0.05 and *P* > 0.21 for CpG-1 and CpG-3). None of the three CpGs were significantly associated with the Trait subscale, with CpG-1 being the most significant (−0.32, *P* = 0.13).

**FIGURE 3:**
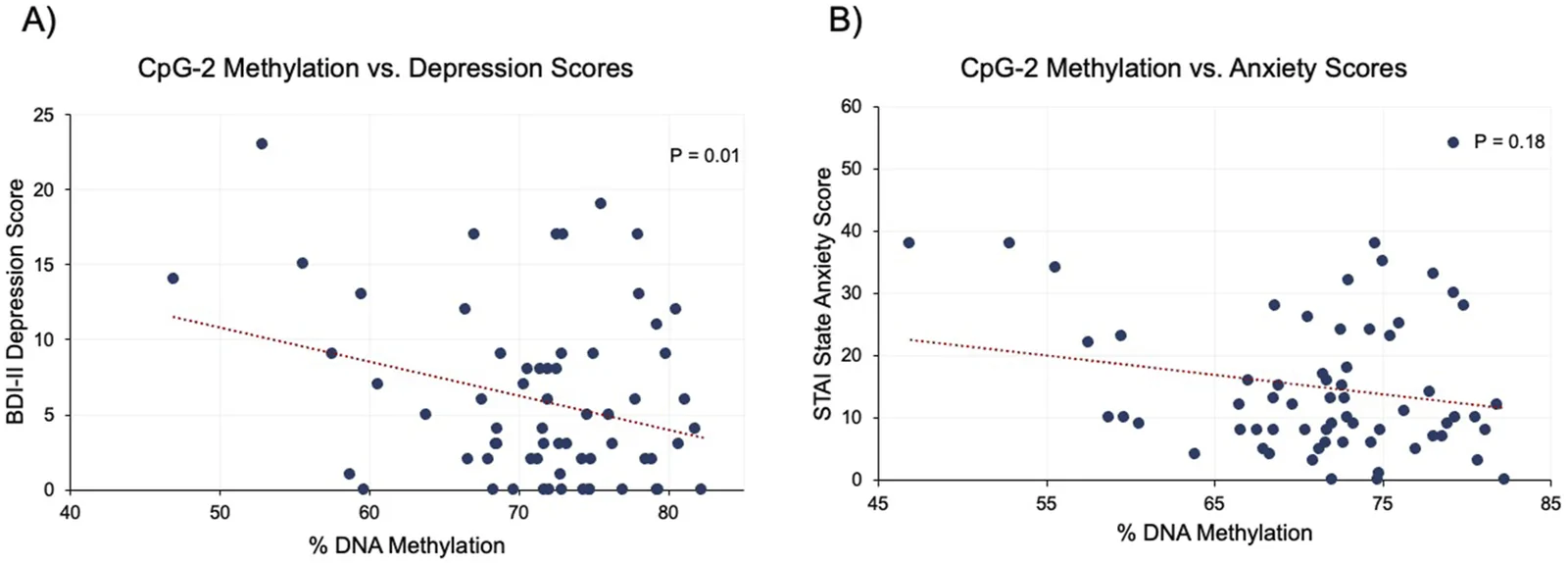
Association between mood and anxiety scores vs. *CRF* methylation at CpG-2. **(A)** Linear regression analysis showed a significant, negative association between CRF methylation and depression scores (BDI-II). **(B)** The association between *CRF* methylation and STAI State scores were non-significant.

### Association between *CRF* methylation and amygdala volume

Given a previous study linking anxiety and depression scores with amygdala volume (Santos et al., 2017), *CRF* methylation was compared to amygdala volume. For all study participants, methylation levels were first compared to total amygdala volume consisting of both left and right regions and then each right or left region separately. Interestingly, we observed significant, positive associations with CpG-1 methylation when it was compared to total (r = 0.37, *P* = 0.003, Figure 4A), right (r = 0.36, *P* = 0.004, Figure 4B), and left (r = 0.34, *P* = 0.006, Figure 4C) amygdala volumes. Methylation levels at CpG-2 and CpG-3 were not correlated with any of the volume metrics (r ≤ 11.92, P ≥ *P*.51). For CpG-1, these relationships remained significant after adjusting for age, sex, and education in all three amygdala volume measurements using linear regression: total (28.20, *P* = 0.*P*06), left (11.87, *P* = 0.02), and right (16.33, P = *P*.005) amygdala volumes (Table 2).

**FIGURE 4:**
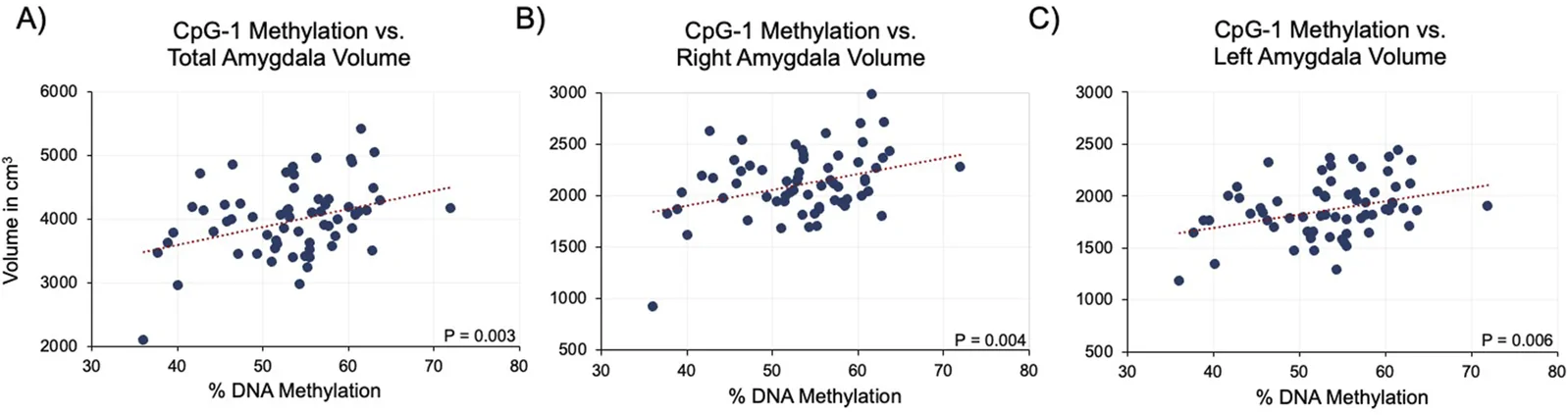
Association between *CRF* methylation at CpG-1 and amygdala volume. Linear regression analysis showed a significant, positive association between *CRF* methylation and total **(A)**, right **(B)**, and left **(C)** amygdala volumes.

### Association between DNA methylation and gene expression

Finally, since only blood DNA samples were available from the CS patients, it was not possible to determine whether the methylation levels of the three CpGs were generally associated with *CRF* gene expression. To demonstrate this association, amygdala tissues from twenty deceased individuals with no diagnosis of psychiatric disorders or CS were examined. Genomic DNA and messenger RNA extracted simultaneously from the same tissue were used for *CRH* methylation analysis and gene expression, respectively. Analysis using Pearson’s coefficient showed a negative correlation between gene expression and methylation at all three CpGs: CpG-1 (−0.62, *P* = 0.004, Figure 5A), CpG-2 (−0.40, *P* = 0.08, Figure 5B), and CpG-3 (−0.53, *P* = 0.02, Figure 5C) with the strongest correlation at CpG-1.

**FIGURE 5:**
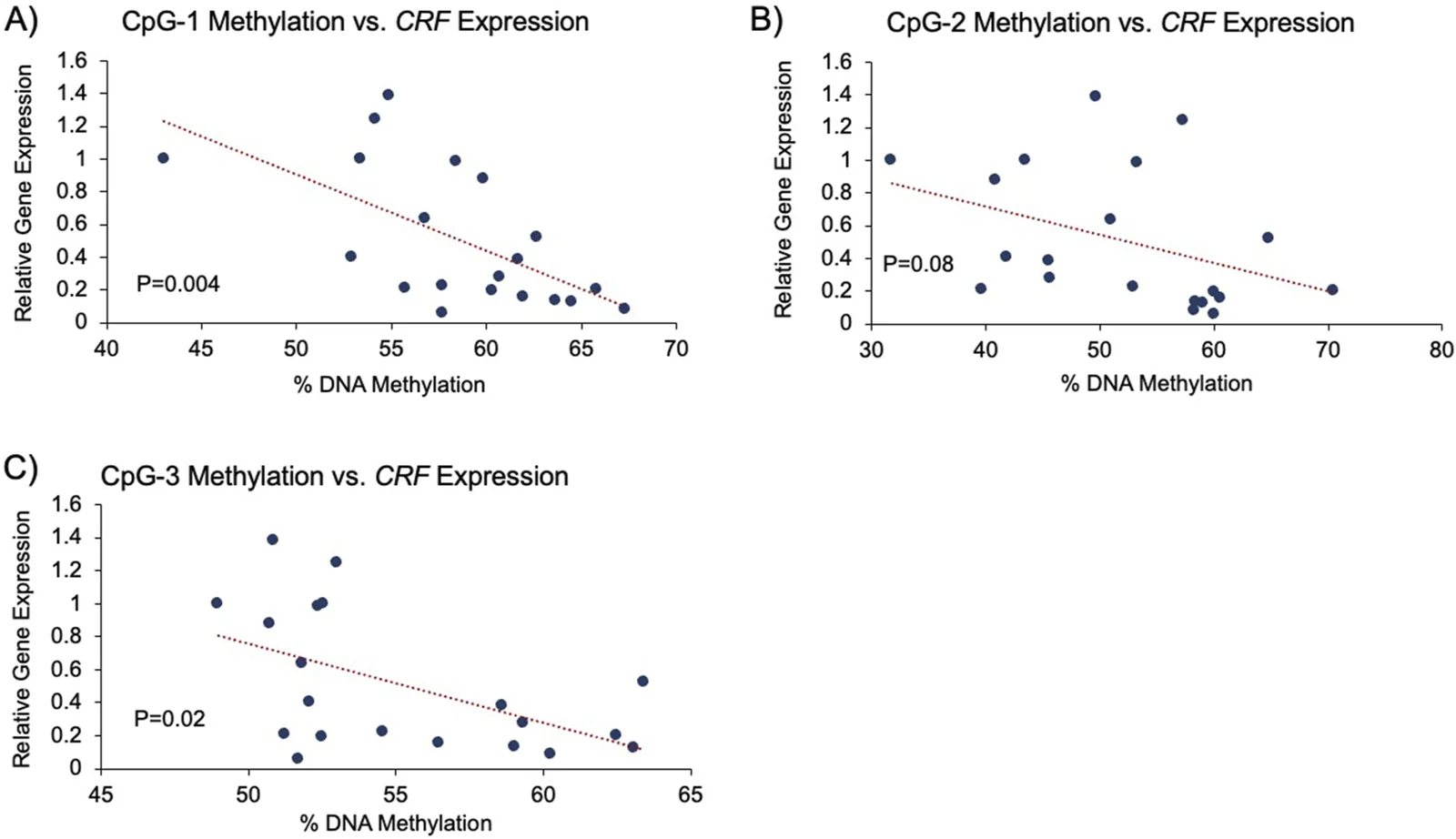
Association between *CRF* expression and promoter methylation. Analysis using Pearson’s correlation coefficient showed a significant, negative correlation between gene expression and DNA methylation in postmortem amygdala tissues of healthy controls. **(A–C)** Pearson’s correlation analysis between CRF expression and CpGs 1-3, respectively, is shown.

## Discussion

In the current study, we explored the relationship among *CRF* promoter CpG methylation, amygdala volume, and mood status in CS patients, aimed at assessing whether DNA from a peripheral tissue such as blood could be used to inform us about the detrimental consequences of chronic hypercortisolemia on the amygdala and mood.

We observed for the first time a consistent decrease in DNA (CpG) methylation levels in CS patients, with healthy controls showing the highest *CRF* methylation levels and cured CS samples showing lower levels, followed by active CS samples showing the lowest. The disease status-dependent decrease in *CRF* methylation is reminiscent of the intronic methylation levels observed at the *FKBP5* locus, which also showed a CS-associated decrease in methylation (Resmini et al., 2016). In both cases, the cured CS group showed methylation levels that were between those of the active CS cases and controls, suggesting that GC-induced loss of methylation in blood may only be partially reversible. Such methylation patterns underscore the durability of GC-induced epigenetic changes and may reflect similarly irreversible CS symptomatology.

Postmortem amygdala tissues from deceased healthy individuals were used to establish a functional role for the *CRF* CpGs by showing that these CpGs may play a role in influencing gene expression by conferring methylation-sensitive binding of transcription factors to the *CRF* promoter. Since leukocyte mRNA was not collected from the CS cohort, it was not possible to assess whether CpG methylation levels associated with *CRF* expression levels. Further, since we had compared methylation levels in blood to volumetric and psychometric measurements associated with the amygdala, we sought to determine whether methylation correlated with gene expression in this relevant tissue. A significant relationship that was observed between *CRF* gene expression and CpG methylation suggests that methylation levels of at least two of the three CpGs likely influence *CRF* expression in the amygdala. All three CpGs tested are conserved across species and located approximately 200 bases downstream of negative GC and cAMP response elements that are thought to mediate cortisol- and stress-induced regulation of the *CRF* gene (Malkoski et al., 1997). Importantly, data from another group demonstrated that these three CpGs underwent significant loss of methylation in mice that were susceptible to stress, and these CpGs conferred methylation-sensitive gene expression in an *in vitro* reporter assay (Elliott et al., 2010). Loss of *CRF* methylation in CS implies that hypercortisolism may lead to increased *CRF* expression in the amygdala. While exposure to excess GCs leads to the downregulation of CRF in the hippocampus and the hypothalamus as part of the negative feedback mechanism of the HPA axis (Lee et al., 2010), there is a positive feedback in the amygdala (Gold and Chrousos, 2002), where GC administration activates *CRF*, especially in the central amygdala (Makino et al., 1999; Shepard et al., 2000; Kolber et al., 2008; Tran and Greenwood-Van Meerveld, 2012). Taken together, our results suggest that methylation of *CRF* promoter could be a functional, peripheral biomarker of mood and amygdala volume in CS patients.

We also examined the association between *CRF* methylation and mood data. While a significant association was observed between BDI-II depression scores and *CRF* methylation, there was no significant association between the STAI anxiety scores and methylation. This finding was surprising given the central role that CRF plays on anxiety in the amygdala. However, CRF and anxiety may be involved in the context of chronic stress rather than in elevated GC exposure alone and thus may involve additional neuroendocrine factors, such as noradrenaline (Tanaka et al., 2000).

Finally, we observed a positive relationship between DNA methylation levels of *CRF* and total amygdala volume. Specifically, higher CpG-1 methylation levels were associated with higher amygdala volumes and *vice versa*, and this association held when left and right amygdala volumes were analyzed separately. Since reduced methylation levels were associated with hypercortisolism and increased *CRF* expression, this finding suggests a potential role for *CRF* expression in amygdala, the volume reduction of which has been previously observed in our CS cohort (Santos et al., 2017) and in patients receiving chronic, exogenous GC administration (Brown et al., 2008). Consistent decrease in amygdala volume was also documented in unmedicated major depression patients in a meta-analysis study (Hamilton et al., 2008). However, association studies of amygdala volume with other stress-related pathologies such as anxiety disorder and PTSD have yielded mixed results, largely owing to differences in age, sex, and nature and duration of the stressors. Considering our findings, additional work is necessary to demonstrate the role of *CRF* expression on amygdala volumes in the context of these other psychopathologies.

Our study has several limitations. The sample size of our cohort is relatively small, but this is difficult to avoid in a rare disease such as CS, especially in cohorts where multidimensional data such as brain volume, psychometric, and cognitive data have been collected. Also, our study population included patients with pituitary-dependent CS and adrenal CS. We did not segregate the two types of CS, since doing so would further reduce the sample size, and they both shared chronic exposure to endogenous hypercortisolism, which we assumed would be detrimental to the brain regardless of CS etiology. Nine subjects with active CS were taking ketoconazole, which could have also led to a potential confound.

In addition, although significant relationships were observed between blood DNA methylation and processes related to the brain, there were several experimental steps in between that could not be performed. Previous work in mice showed that treatment with high-dose GCs caused similar methylation changes in both blood and brain tissues (Ewald et al., 2014; Seifuddin et al., 2017). In these studies, the use of an animal model enabled the investigation of GC-treated blood and brain tissues from the same animals so that biologically meaningful correlations could be derived between the two tissues. In the current clinical cohort, while an attempt was made to demonstrate that the same set of CpGs in the amygdala were associated with gene expression, the question of whether CS-associated methylation levels in the blood correlated with methylation changes in the amygdala remains undetermined. To answer this question, both blood and brain tissues from the same patients are needed to elucidate this relationship, which is not feasible in living CS patients. We also acknowledge that unlike the significant relationship that we observed between *CRF* methylation and gene expression in the control postmortem brains, there may be additional factors and mechanisms that can contribute to *CRF* expression in CS patients.

Another limitation was the inability to distinguish epigenetic changes that reflect a reduction in DNA methylation from those which may be due to changes in the cellular composition of blood. During chronic exposure to high-dose GCs, there occurs a substantial shift in the cellular proportion of blood. For instance, when mice are exposed to high-dose GCs, neutrophils assume a greater percentage of circulating white blood cells in a process known as demargination (Seifuddin et al., 2017). Since each cell type exhibits distinct methylation patterns across its genome, GC-induced changes in methylation may also capture the increasing contribution of methylation content from a cell type, such as those from neutrophils that increases in proportion in the GC-exposed blood. While there are several statistical methods for estimating cell type proportion changes based on unique methylation signature of each cell type, these methods rely on the use of genome-wide methylation platforms such as the Illumina 450K or EPIC arrays, with hundreds of CpG probes being used for each cell-type signature (Houseman et al., 2012). We acknowledge that our analysis of *CRF* precluded the use of such an array. However, we note that a whole blood cell count performed in cured CS patients in this cohort showed similar percentage of neutrophil and lymphocyte counts as those of the controls (Aulinas et al., 2014). This implies that for at least the cured CS samples, none of the analysis that involves DNA methylation are likely to be due to a shift in the cellular composition of blood. Finally, we acknowledge the relatively small effect size in DNA methylation differences between CS and controls. We observed a bigger effect size when testing GC-induced DNA methylation changes in the mouse blood. However, the bigger effect size in mice was due to the combination of demargination and loss of DNA methylation in the post GC-treatment period that was not as prominent in human CS (Seifuddin et al., 2017). We speculate that methylation differences in CS blood may be smaller due, in part, to the smaller percentage of lymphocytes in humans (30–50%) compared to mice (75–90%) (Doeing et al., 2003; Mestas and Hughes, 2004). A previous study by our group has shown that GC-induced loss of methylation occurs primarily in T-cells, implying that mice with a larger percentage of T-cells in the blood than humans will show greater changes in DNA methylation (Seifuddin et al., 2017). Nevertheless, we observed significant correlations between CRF methylation and CS status, BDI-II scores, and amygdala volume, underscoring the unlikelihood that these methylation differences are spurious findings. In addition, our effect sizes are similar to or greater than those (methylation M-values) obtained from a recent methylomic study of Cushing’s disease using the EPIC methylation platform (Armignacco et al., 2022).

Despite these limitations, our study shows that epigenetic patterns at the *CRF* locus could be associated with mood and amygdala volume alterations in CS patients. Our findings support the feasibility of using epigenetic patterns in blood as a surrogate for assessing GC-related pathologies in the brain.

## Data Availability

The datasets presented in the study are publicly available. This data can be found here: https://figshare.com/articles/dataset/_b_Blood_DNA_methylation_of_b_b_b_b_i_CRF_i_b_b_b_b_and_its_association_with_amygdala_volume_and_mood_in_Cushing_s_syndrome_b_/27084100?file=49350520, DOI: https://10.6084/m9.figshare.27084100.

## Abbreviations

BDI, Beck Depression Inventory; CRF, human protein; CRF, human gene; CRF, mouse protein; *Crf*, mouse gene; FKBP5 FK506, Binding Protein 5; PTSD post-traumatic stress disorder; STAI, State-Trait Anxiety Inventory.
